# BUB1 promotes proliferation of liver cancer cells by activating SMAD2 phosphorylation

**DOI:** 10.3892/ol.2020.11445

**Published:** 2020-03-05

**Authors:** Li-Jing Zhu, Yan Pan, Xiao-Ying Chen, Pan-Fei Hou

**Affiliations:** 1 Department of Radiation Oncology, Lianshui County People's Hospital, Huaian, Jiangsu 223400, P.R. China; 2 Department of Clinical Laboratory, Lianshui County People's Hospital, Huaian, Jiangsu 223400, P.R. China; 3 Clinical Laboratory, Ren Ji Hospital, School of Medicine, Shanghai Jiao Tong University, Shanghai 200127, P.R. China

**Keywords:** budding uninhibited by benzimidazoles 1, proliferation, molecular target, liver cancer, TGF-β/SMAD

## Abstract

Budding uninhibited by benzimidazoles 1 (BUB1) is a mitotic checkpoint serine/threonine kinase that has been reported as an oncogene or tumor suppressor gene in various types of cancer, including breast cancer, pancreatic ductal adenocarcinoma, prostate and gastric cancers. However, its role in liver cancer remains unclear. The present study aimed to explore the biological function of BUB1 in liver cancer. The present study demonstrated that BUB1 mRNA expression levels and the intensity of immunohistochemical staining were significantly increased in liver cancer tissues compared with normal tissues. The role of BUB1 in cell proliferation was also determined. Overexpression of BUB1 significantly promoted cell proliferation, whereas knockdown of BUB1 expression inhibited the proliferation of liver cancer cell lines. In experiments investigating the underlying mechanism, overexpression of BUB1 increased the levels of SMAD2 phosphorylation, whereas knockdown of BUB1 reduced the levels of SMAD2 phosphorylation. Therefore, BUB1 may promote proliferation of liver cancer cells by activating phosphorylation of SMAD2, and BUB1 may serve as a potential target in the diagnosis and/or treatment of liver cancer.

## Introduction

Liver cancer is one of the most common malignant tumors with high morbidity and mortality rates, with 854,000 new cases and 810,000 mortality cases per year (1). Liver cancer is the fifth most common cancer and the second leading cause of cancer-associated mortality worldwide in 2018 (2). Multiple treatment options are available for patients with liver cancer, including surgical resection, transarterial chemoembolization, radiotherapy and sorafenib (3). However, the prognosis of patients with liver cancer after surgery remains poor due to the postoperative recurrence and early blood vessel invasion (4,5). Liver cancer has been demonstrated to be associated with mutations of numerous genes, including catenin beta 1, tumor protein p53 and axin 1 (6). Novel molecular and cellular targets, including cancer stem wells, were identified, allowing the development of a novel therapy for patients with advanced liver cancer, resulting in favorable curative effects and significantly prolonging the patients' survival time (7). Since preliminary progress has been made in the molecular-targeted therapy of liver cancer, the present study will determine potential novel biomarker for the early diagnosis of liver cancer and the prognosis of patients.

Budding uninhibited by benzimidazoles 1 (BUB1) is a mitotic checkpoint serine/threonine kinase that serves a central role in aligning chromosomes and establishing the mitotic spindle checkpoint (8). In addition, BUB1 also serves an important role in the accurate partitioning of chromosomes during the cleavage of daughter cells from mother cells (9,10). BUB1 contains three primary regions: A conserved N-terminal region containing the kinetochore localization domain; an intermediate, non-conserved region that acts as a scaffold for the recruitment of proteins; and a C-terminal region that contains a catalytic serine/threonine kinase domain (11). The function of BUB1 as oncogene or tumor suppressor gene has been observed in various types of cancer, including breast cancer, pancreatic ductal adenocarcinoma, prostate and gastric cancer (12–15). Several studies have demonstrated the unfavorable prognostic role of BUB1 in liver cancer based on bioinformatics analysis (16–18). However, the molecular biological function of BUB1 in liver cancer still remains unclear.

In the present study, the importance of BUB1 in the progression of liver cancer was investigated. Initially, reverse transcription-quantitative (RT-q)PCR and immunohistochemistry were used to determine the expression of BUB1 in liver cancer and adjacent normal tissues. In addition, the significance of BUB1 in tumor cell proliferation was demonstrated *in vitro* and the molecular mechanism underlying BUB1 function in liver cancer growth was evaluated.

## Materials and methods

### 

#### Liver cancer tissue samples

A total of 24 pairs of primary liver cancer tissues and their corresponding adjacent normal tissues were obtained from patients who underwent hepatectomy between February 2002 and July 2012 at the Lianshui County People's Hospital (Huaian, China). The median age of patients was 54 years, and there were 18 men and 6 women. The inclusion criteria were as follows: i) Patients clinically diagnosed with liver cancer following surgery; ii) R0 resection of all patients based on histologic examinations, and iii) paired normal tissue was adjacent to tumor tissue with distance <2 cm. The exclusion criteria were as follows: i) Patients with distant metastasis and ii) patients who had received radiotherapy or chemotherapy before surgery. The present study was approved by the Institutional Review Board of The Institute for Lianshui County People's Hospital. Written informed consent was obtained from all patients prior to enrollment. The 24 paired samples were subjected to RNA extraction for RT-qPCR.

#### Microarray data

The relative mRNA expression levels of BUB1 in liver tumor tissues and their corresponding adjacent normal tissues was obtained from The Cancer Genome Atlas (TCGA) database (https://portal.gdc.cancer.gov/). Survival curves of patients with liver cancer stratified according to the median expression levels of BUB1 were also obtained from TGCA.

#### Immunohistochemistry

Clinical liver cancer tissues and paired non-cancerous tissues were fixed in formalin at room temperature for 24 h, embedded in paraffin and cut into 5-µm consecutive sections. Following deparaffinization and antigen recovery in a sodium citrate solution (pH 6.0) for 20 min at 98°C, the sections were washed thrice with 0.01 mol/l PBS for 5 min each time, blocked for 1 h in 0.01 mol/l PBS containing 0.3% Triton X-100 (Santa Cruz Biotechnology, Inc.) and 5% BSA (Gibco; Thermo Fisher Scientific, Inc.), and incubated with an anti-BUB1 antibody (cat. no. ab195268; 1:200; Abcam) overnight at 4°C. Following washing with 0.01 mol/l PBS, the sections were incubated with 0.01 mol/l PBS containing a horseradish peroxidase-conjugated anti-rabbit immunoglobulin G antibody (cat. no. ab6759; 1:500; Abcam) for 2 h at room temperature, followed by development with 0.003% H_2_O_2_ and 0.03% 3,3′-diaminobenzidine in 0.05 mol/l Tris-HCl (pH 7.6). The categories and percentages of immunohistochemical stained cells were assessed in five independent high-power microscopic fields for each tissue sample using a light microscope (magnification, ×400).

#### RNA extraction and RT-qPCR

The specimens were snap-frozen in liquid nitrogen and stored at −80°C use. The total RNA of tumor tissues and adjacent noncancerous tissues from the 24 patients was isolated using TRIzol^®^ reagent (Invitrogen; Thermo Fisher Scientific, Inc.) and reverse-transcribed to cDNA using the PrimeScript RT reagent kit (Takara Bio, Inc.) according to the manufacturer's instructions. SYBR^®^ Premix Ex Taq (Takara Bio, Inc.) was used for qPCR. The thermocycling conditions used for the PCR were as follows: 95°C for 1 min; 40 cycles of 95°C for 12 sec and 58.5°C for 40 sec. The primers were as follows: BUB1 forward, 5′-TGGGAAAGATACATAAGTGGGT-3′ and reverse, 5′-AGGGGATGACAGGGTTCCAAT-3′; GAPDH forward, 5′-ATGACCCCTTCATTGACCTCA-3′ and reverse, 5′-GAGATGATCACCCTTTTGGCT-3′. GAPDH was used as the internal control. The relative mRNA expression level of BUB1 in each sample was calculated using the comparative expression level 2−^∆∆Cq^ method (19).

#### Cell culture

Liver cancer cell lines YY-8103, MHCC97-L, HepG2, and Huh7 were purchased from The Cell Bank of The Type Culture Collection of Chinese Academy of Sciences, and all cell lines were authenticated by STR profiling. Cells were maintained in a humidified atmosphere containing 5% CO_2_ at 37°C in DMEM (Invitrogen; Thermo Fisher Scientific, Inc.) supplemented with 100 U/ml penicillin, 100 mg/ml streptomycin and 10% FBS (both Invitrogen; Thermo Fisher Scientific, Inc.).

#### Cell transfection

The full-length cDNA encoding human BUB1 was obtained from human whole blood by RT-PCR. The human BUB1 gene primer pair was designed using Primer version 5 (PREMIER Biosoft). BUB1 cDNA was cloned into a p23-3×flag-GFP vector (Takara Bio, Inc.) according to the manufacturer's instructions. Lentiviral supernatants were produced using the Lenti-X HTX packaging system (Clontech Laboratories, Inc.) and used for transduction of YY-8103 and MHCC97-L cell lines. For negative controls, cell lines were transduced with supernatants from empty vector cells. The fluorescence and infection efficiency were determined using an inverted fluorescence microscope by GFP sorting (magnification, ×200; IX-71; Olympus Corporation). Over-expressed BUB1 with a Flag tag was detected in cell lines with an anti-Flag (cat. no. 8164; 1:1,000; Cell Signaling Technology, Inc.).

shRNA plasmids for BUB1, which were designed against the BUB1 gene and constructed in Phblv-u6-puro vectors, were purchased from Shanghai GenePharma Co., Ltd. A non-target scrambled oligonucleotide served as the negative control (shcontrol; Shanghai GenePharma Co., Ltd.). All plasmids were verified by sequencing. To generate stable BUB1-silenced cell lines, HepG2 and Huh7 cells were cultured in 6-well plates until they reached 40% confluence. The medium was then replaced with 1 ml fresh FBS-free culture medium supplemented with 40 µl viral supernatant (1×10^8^ UT/ml) and 6 µg/ml polybrene (Han Heng Biotechnology Co., Ltd.) for 24 h. Cells were cultured and screened in medium containing 2.5 µg/ml puromycin (Han Heng Biotechnology Co., Ltd.). Individual puromycin-resistant colonies were isolated during drug screening. The knockdown efficiency was verified by western blotting. The shRNA sequences used in the present study were as follows: shBUB1, forward 5′-CCGGGAATTTCAATTGGGTTCTAAGCTCGAGCTTAGAACCCAATTGAAATTCTTTTTG-3′, reserve 5′-AATTCAAAAAGAATTTCAATTGGGTTCTAAGCTCGAGCTTAGAACCCAATTGAAATTC-3′; and shcontrol, forward 5′-CCGGCAAACTTTGTATGCCCGCTTTCTCGAGAAAGCGGGCATACAAAGTTTGTTTTTG-3′ and reserve 5′-AATTCAAAAACAAACTTTGTATGCCCGCTTTCTCGAGAAAGCGGGCATACAAAGTTTG-3′.

#### Western blot analysis

Cells were lysed in RIPA Lysis Buffer and phenylmethylsulfonyl fluoride (PMSF) (Thermo Fisher Scientific, Inc.) and the lysates were centrifuged at 10,000 × g for 15 min at 4°C according to the manufacturer's protocols. Protein concentration was determined using Bradford reagent (Sigma-Aldrich; Merck KGaA). Proteins (15 µg) were separated by 10% SDS-PAGE and transferred onto nitrocellulose membranes (EMD Millipore). Membranes were blocked with 5% fat-free milk for 1 h at room temperature and incubated with primary antibodies against BUB1 antibody (cat. no. ab195268; 1:1,000; Abcam), anti-SMAD2 (cat. no. 5339; 1:1,000; Cell Signaling Technology, Inc.), phospho- (p) SMAD2 (cat. no. 3104; 1:1,000; Cell Signaling Technology, Inc.), PCNA (cat. no. 13110; 1:1,000 dilution; Cell Signaling Technology, Inc.), Ki67 (cat. no. 2586; 1:1,000; Cell Signaling Technology, Inc.), Flag (cat. no. 8164; 1:1,000; Cell Signaling Technology, Inc.) and GAPDH (cat. no. 5174; 1:1,000; Cell Signaling Technology, Inc.) at 4°C overnight. Membranes were then incubated with anti-rabbit horseradish peroxidase-conjugated secondary antibody (cat. no. 7074; 1:5,000; Cell Signaling Technology, Inc.) for 2 h at room temperature. The immunoreactive protein bands were visualized using an enhanced chemiluminescence kit (Pierce; Thermo Fisher Scientific, Inc.) and a Gel Dox XR system (Bio-Rad Laboratories, Inc.).

#### Crystal violet assay

A total of 1×103 cells/well were seeded into 6-well plates and the cells were cultured in medium with 10% FBS. The medium was changed every three days. After two weeks, the medium was removed and cells were fixed with 20% methanol at room temperature for 10 min and stained with 0.5% crystal violet (Sigma-Aldrich; MerckKGaA). Subsequently, cells were washed with PBS and images were captured using digital camera. Then, 1 ml glacial acetic acid was added to the cells, and the optical density (OD) was detected at 600 nm using a microplate reader.

#### MTT assay

A total of 1×103 cells/well were seeded into 96-well plates, and cell viability was detected using MTT. After 1, 2, 3, 4, 5, 6, or 7 days of incubation, 20 µl 5 mg/ml MTT was added to each well and incubated at 37°C for a further 4 h. Subsequently, the medium was aspirated and the wells washed with PBS and drained for ~2 h. Any remaining solution was carefully aspirated and 200 µl DMSO was added to dissolve the formazan crystal with gentle agitation. The optical density was measured at 490 nm using a microplate reader.

#### Statistical analysis

Statistical evaluations were performed using GraphPad Prism 5 (GraphPad Software Inc.), and the data are presented as mean ± standard deviation unless otherwise stated. Cell proliferation rates were compared using an unpaired Student's t-test. P<0.05 was considered to indicate a statistically significant difference.

## Results

### 

#### BUB1 expression is upregulated in liver cancer tissues

**Figure 1.**
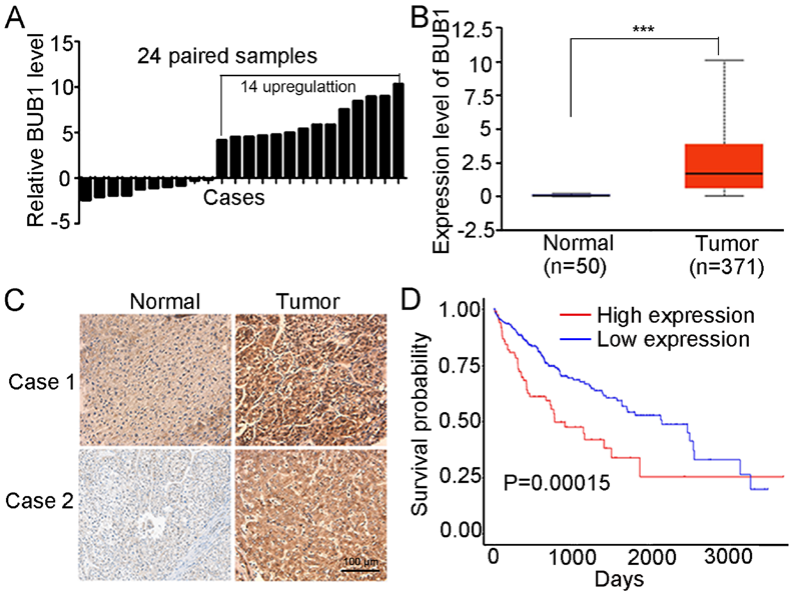
BUB1 expression is high in liver cancer tissues and is associated with the survival of patients with liver cancer. (A) BUB1 mRNA expression levels in 24 pairs of tumor samples relative to matched normal hepatic tissues. (B) Expression levels of BUB1 were higher in tumor tissues compared with normal tissues based on data obtained from The Cancer Genome Atlas. (C) Immunohistochemistry staining of BUB1 in paired normal and tumor tissues from two patients. Magnification, ×400. (D) High BUB1 expression levels were associated with poor overall survival time in patients with liver cancer. ***P<0.001. BUB1, budding uninhibited by benzimidazole 1.

The mRNA expression levels of BUB1 in 24 pairs of liver cancer tissues and the corresponding normal tissue were examined. The results of the RT-qPCR demonstrated that mRNA expression of BUB1 was significantly higher in 14 of the tumor samples compared with the matched normal tissues (Fig. 1A). Based on the obtained data from TCGA, BUB1 expression was significantly higher in 371 tumor tissues compared with 50 normal tissues (P<0.001; Fig. 1B). Immunohistochemistry analysis revealed that BUB1 was primarily expressed in the cytoplasm, and the tumor tissues exhibited increased staining intensity compared with the paired normal tissues in five patients (Figs. 1C and S1), consistent with the results of RT-q PCR. In addition, the overall survival rates were significantly higher in the BUB1-high group compared with the BUB1-low group (P <0.001; Fig. 1D). These results suggested that BUB1 was upregulated in liver cancer tissues.

#### Overexpression and knockdown of BUB1 in liver cancer cell lines

**Figure 2.**
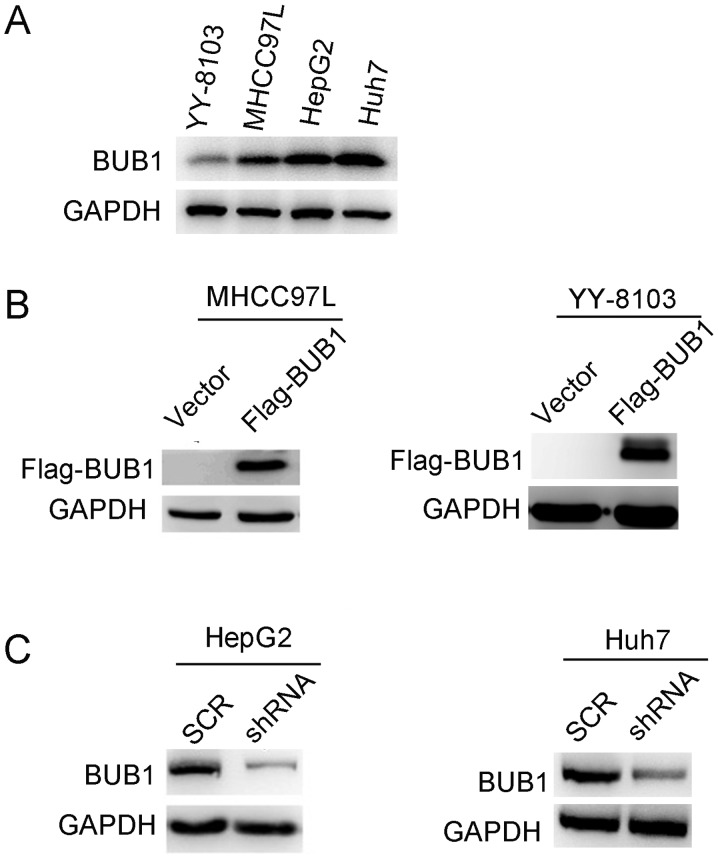
Overexpression and knockdown of BUB1 in liver cancer cell lines. (A) Western blots of BUB1 expression in four liver cancer cell lines: YY-8103, MHCC97-L, HepG2 and Huh7. GAPDH was used as the loading control. (B) Western blots of overexpression of BUB1 in transfected MHCC97-L and YY-8103 cells. (C) Western blots of knockdown of BUB1 in HepG2 and Huh7 cells. BUB1, budding uninhibited by benzimidazole 1; SCR, scrambled; sh, short hairpin.

Based on the clinical data, it was hypothesized that BUB1 may promote the proliferation of liver cancer cells. To determine the effects of BUB1, the expression levels were determined in several liver cancer cell lines. The results demonstrated that BUB1 protein expression levels were higher in HepG2 and Huh7 cell lines compared with YY-8103 and MHCC97-L cells (Fig. 2A). To determine the effects of BUB1 in liver cancer cells, the MHCC97-L and YY-8103 cells were transfected with plasmids containing either an empty p23 vector or BUB1 overexpression vectors (Flag-BUB1), whereas shRNA targeting BUB1 was transfected into HepG2 and Huh7 cells. Western blotting results revealed that the establishment of overexpression and knockdown of BUB1 in liver cancer cell lines was successful (Fig. 2B and C).

#### BUB1 overexpression promotes the proliferation of the liver cancer cells

**Figure 3.**
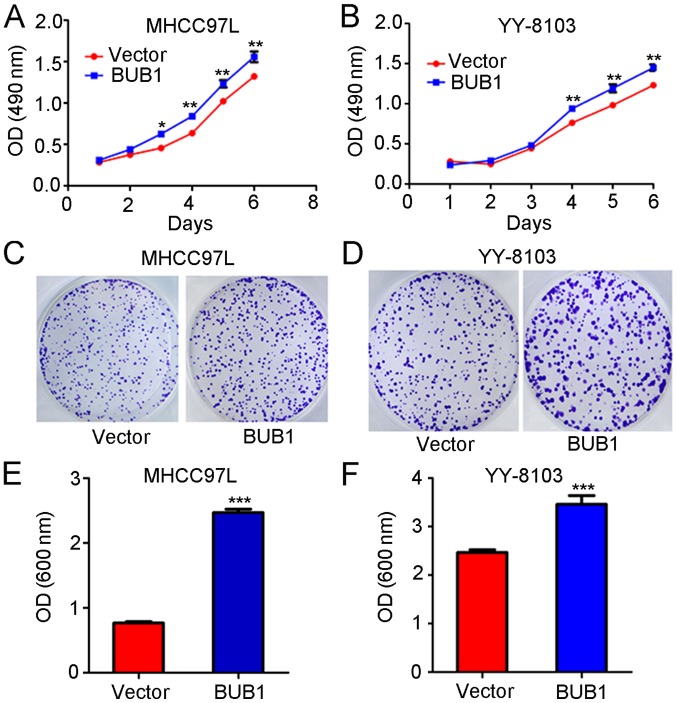
BUB1 overexpression increases liver cancer cell proliferation. The effects of BUB1 overexpression on the viability of (A) MHCC97-L and (B) YY-8103 cells were assessed. The effects of BUB1 overexpression on the viability of (C) MHCC97-L and (D) YY-8103 cells were assessed using crystal violet assays. The OD values of the crystal violet assay in (E) MHCC97-L and (F) YY-8103 cells. *P<0.05, **P<0.01 and ***P<0.001 vs. vector. BUB1, budding uninhibited by benzimidazole 1; OD, optical density.

MTT assay revealed that the absorbance values of the MHCC97-L cells 3, 4, 5 and 6 days after transfection with the BUB1 overexpression vector were significantly higher compared with untreated cells (P<0.01; Fig. 3A). Similarly, the absorbance values of YY-8103 cells at 4, 5 and 6 days after transfection with the BUB1 overexpression vector were significantly increased compared with the untreated cells (P<0.01; Fig. 3B). The results of the crystal violet assay demonstrated that the absorbance values of MHCC97-L and YY-8103 cells following transfection with the BUB1 overexpression vector were significantly higher compared with untreated cells (all P<0.01; Fig. 3C-F).

#### BUB1 knockdown inhibits the proliferation of liver cancer cells

**Figure 4.**
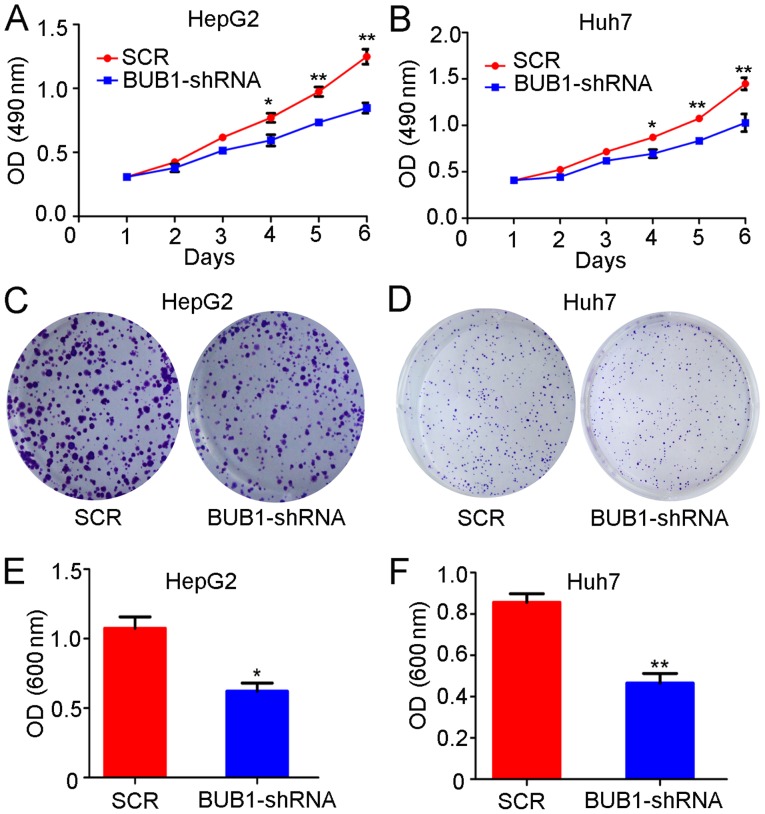
BUB1 knockdown inhibits liver cancer cell proliferation. The effects of BUB1 knockdown on the viability of (A) HepG2 and (B) Huh7 cells were assessed. The effects of BUB1 knockdown on the viability of (C) HepG2 and (D) Huh7 cells were assessed using a crystal violet assay. The OD values of the crystal violet assay in (E) HepG2 and (F) Huh7 cells. *P<0.05 and **P<0.01 vs SCR. BUB1, budding uninhibited by benzimidazole 1; OD, optical density; SCR, scrambled; sh, short hairpin.

Similar to the overexpression experiments, the proliferation of the control and BUB1-shRNA cell lines was tested. The results of the MTT assay demonstrated that the absorbance values of HepG2 cells 3, 4, 5 and 6 days after BUB1 knockdown were significantly lower compared with those of untreated cells (P<0.01; Fig. 4A). Similarly, the absorbance values of Huh-7 cells 4, 5 and 6 days after BUB1 knockdown were also significantly lower compared with untreated cells (P<0.01; Fig. 4B). In addition, the crystal violet assay demonstrated that the absorbance of HepG2 (P<0.05; Fig. 4C and E) and Huh-7 cells (P<0.01; Fig. 4D and F) after BUB1 knockdown was significantly lower compared with untreated cells.

#### BUB1 activates the phosphorylation of SMAD2 in liver cancer cells

**Figure 5.**
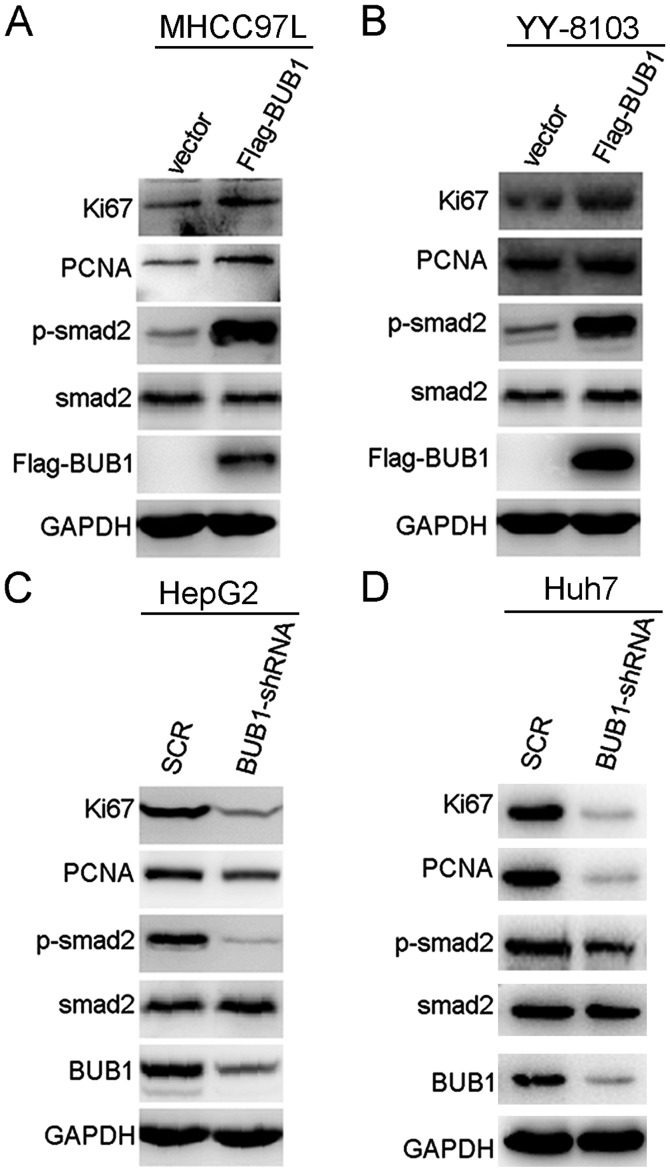
BUB1 activates the TGF-β/SMAD signaling pathway. (A and B) Overexpression of BUB1 increased the expression levels of p-smad2, Ki67, and PCNA in (A) MHCC97-L and (B) YY-8103 cells. (C and D) Knockdown of BUB1 reduced the expression levels of p-smad2, Ki67, and PCNA in (C) HepG2 and (D)Huh7 cells. BUB1, budding uninhibited by benzimidazole 1; OD, optical density; SCR, scrambled; sh, short hairpin; p-, phosphorylated.

To explore the molecular mechanism by which BUB1 affected liver cancer, p-SMAD2 and total SMAD2 protein expression levels in BUB1-overexpressing and knockdown cell lines were measured. Expression levels of p-SMAD2 were increased when BUB1 was overexpressed (Fig. 5A and B). The reverse was observed in HepG2 and Huh-7 cells; knockdown of BUB1 decreased the expression levels of p-SMAD2 (Fig. 5C and D). In addition, BUB1 overexpression notably increased the expression levels of cell proliferation markers Ki67 and PCNA, whereas BUB1 knockdown decreased the expression levels of Ki67 and PCNA (Fig. 5C and D). As demonstrated in Fig. S2, further knockdown of BUB1 in the BUB1-overexpressing MHCC97-L and YY-8103 cells decreased the expression levels of p-SMAD2, Ki67 and PCNA.

## Discussion

Aberrant expression and mutations in BUB1 are associated with aneuploidy and several types of cancer, including breast cancer and pancreatic ductal adenocarcinoma (20). To date, several studies have demonstrated that BUB1 is significantly upregulated in various types of cancer, such as breast cancer, pancreatic ductal adenocarcinoma, prostate and gastric cancer (12–14), and is associated with unfavorable outcomes. However, BUB1 has been reported to serve differing roles in different types of cancer. In endometrial carcinoma (21), low-grade breast cancer (22), and gastric adenocarcinoma (15), high expression levels of BUB1 were associated with a good prognosis, whereas in invasive breast cancer (23) and ovarian cancer (24), high expression of BUB1 was associated with an unfavorable prognosis.

The results of the present study demonstrated that BUB1 mRNA and protein expression levels were significantly increased in liver cancer tissues compared with normal tissues. In addition, western blotting confirmed successful overexpression and knockdown of BUB1 in liver cancer cell lines. It was observed that cell proliferation was significantly increased following BUB1 overexpression, whereas knockdown of BUB1 inhibited liver cancer cell proliferation. Expression levels of p-SMAD2 were significantly increased when BUB1 was overexpressed, whereas knockdown of BUB1 decreased the expression levels of p-SMAD2. The present study demonstrated that BUB1 may promote liver cancer cell proliferation by activating the phosphorylation of SMAD2. It has been reported that constitutive activation of the TGF-β/SMAD signaling pathway serves a crucial role in the development and progression of liver cancer (25). TGF-β exerts its effect on gene expression via interaction with SMAD protein transcription factors, including SMAD2 and SMAD3, followed by R-SMAD and a common mediator SMAD heterodimer formation (26,27). Overactivation of TGF-β signaling serves a complicated role in the development and progression of a range of diseases, including Parkinson's disease and cardiovascular diseases, which is associated with increased growth and invasion at later stages of tumor progression (28,29). To the best of our knowledge, the present study is the first to demonstrate the involvement of BUB1 in the proliferation of liver cancer.

In summary, the present study demonstrated that BUB1 increased the proliferation of liver cancer cells. These results provided an improved understanding of the mechanisms for the role of BUB1 in tumor development and may highlight BUB1 as a potential target in the diagnosis and/or treatment of liver cancer.

## Supplementary Material

Supporting Data

## Data Availability

All data generated or analyzed during the present study are included in this published article.
