# Supplementary material for: BUB1 promotes proliferation of liver cancer cells by activating SMAD2 phosphorylation
Source: Oncol Lett. 2020 Mar 5;19(5):3506–12. doi: 10.3892/ol.2020.11445 (PMC7114935; doi:10.3892/ol.2020.11445)

Figure S1. Immunohistochemistry staining of BUB1 in paired normal and liver tumor tissues from three different patients. Magnification, x400. BUB1, budding uninhibited by benzimidazole 1.

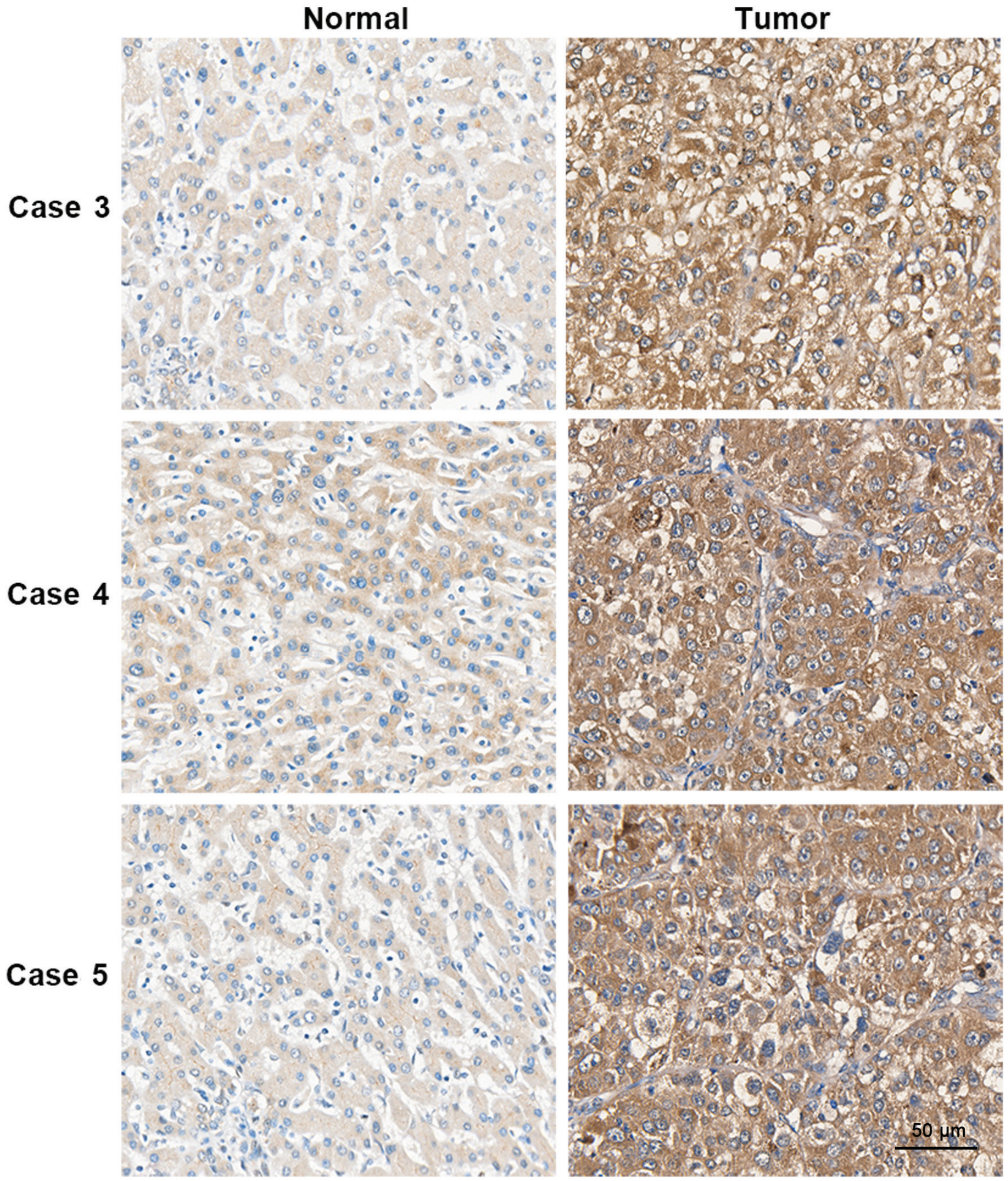

Figure S2. Further knockdown of BUB1 decreases the expression levels of p-SMAD2, Ki67, and PCNA in MHCC97-L and YY-8103 cells overexpressing. BUB1, budding uninhibited by benzimidazole 1; p-, phosphorylated; sh, short hairpin.

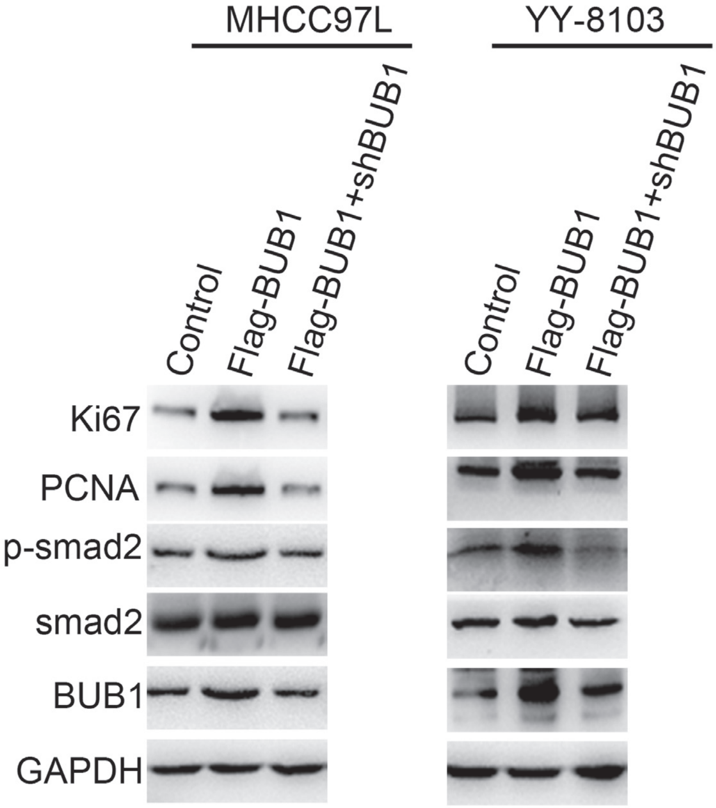

Supplement: Supporting Data [file Supplementary_Data.pdf]
